# Biomimetic Scaffolds Based on Mn^2+^-, Mg^2+^-, and Sr^2+^-Substituted Calcium Phosphates Derived from Natural Sources and Polycaprolactone

**DOI:** 10.3390/biomimetics9010030

**Published:** 2024-01-04

**Authors:** Leonard Bauer, Maja Antunović, Hrvoje Ivanković, Marica Ivanković

**Affiliations:** Faculty of Chemical Engineering and Technology, University of Zagreb, Trg Marka Marulića 19, HR-10001 Zagreb, Croatia; lbauer@fkit.unizg.hr (L.B.); maja.antunovic2007@gmail.com (M.A.); hivan@fkit.unizg.hr (H.I.)

**Keywords:** scaffolds, calcium phosphates, ion substitution, Mn, Mg, Sr, polycaprolactone

## Abstract

The occurrence of bone disorders is steadily increasing worldwide. Bone tissue engineering (BTE) has emerged as a promising alternative to conventional treatments of bone defects, developing bone scaffolds capable of promoting bone regeneration. In this research, biomimetic scaffolds based on ion-substituted calcium phosphates, derived from cuttlefish bone, were prepared using a hydrothermal method. To synthesize Mn^2+^-substituted scaffolds, three different manganese concentrations (corresponding to 1, 2.5, and 5 mol% Mn substitutions for Ca into hydroxyapatite) were used. Also, syntheses with the simultaneous addition of an equimolar amount (1 mol%) of two (Mg^2+^ and Sr^2+^) or three ions (Mn^2+^, Mg^2+^, and Sr^2+^) were performed. A chemical, structural, and morphological characterization was carried out using X-ray diffraction, Fourier transform infrared spectroscopy, and scanning electron microscopy. The effects of the ion substitutions on the lattice parameters, crystallite sizes, and fractions of the detected phases were discussed. Multi-substituted (Mn^2+^, Mg^2+^, and Sr^2+^) scaffolds were coated with polycaprolactone (PCL) using simple vacuum impregnation. The differentiation of human mesenchymal stem cells (hMSCs), cultured on the PCL-coated scaffold, was evaluated using histology, immunohistochemistry, and reverse transcription–quantitative polymerase chain reaction analyses. The expression of collagen I, alkaline phosphatase, and dentin matrix protein 1 was detected. The influence of PCL coating on hMSCs behavior is discussed.

## 1. Introduction

The treatment of bone defects caused by trauma, disease, or tumor resection represents a significant medical challenge. Bone is the second most frequently transplanted tissue in the world [1]. The conventional use of bone grafts (autografts, allografts, or xenografts) in the reconstruction of bone defects has several disadvantages such as the presence of a second surgical site, limited donor availability, possible immunological reaction, and disease transmission. To address these limitations, bone tissue engineering (BTE) has appeared as a promising approach, developing bioactive and biodegradable 3D porous scaffolds and acting as artificial extracellular matrices (ECMs) that support bone regeneration [2].

An ideal scaffold is a highly porous material with interconnected pores that enables the transport of nutrients, oxygen, and metabolic waste products and support vascularization. Its surface properties must support cell attachment, proliferation, and differentiation. Also, the scaffold should be bioresorbable, with a controllable degradation rate that corresponds to the rate of new bone formation. Additionally, the scaffold should also have appropriate mechanical properties.

In recent decades, hydroxyapatite (HAp, Ca_10_(PO_4_)_6_(OH)_2_) bioceramics have received considerable attention as bone graft materials [3]. Bone mineral is a non-stoichiometric form of hydroxyapatite, with a Ca/P ratio less than 1.67, substituted with trace ions such as Na^+^, Sr^2+^, Mg^2+^, Zn^2+^, Mn^2+^, SiO_4_^4−^, CO_3_^2–^, etc. [4].

There have been many studies demonstrating the importance of trace elements in bone formation, and up to now, significant attention has been paid to ionic substitutions into hydroxyapatite lattices [5]. For example, the bivalent cations Mg^2+^, Mn^2+^, and Sr^2+^ are reported to stimulate osteoblast activity, and their deficiency may result in osteoporosis and other bone tissue abnormalities [6,7,8,9]. Magnesium is one of the main substituents in biological apatites (0.44 wt.% in enamel, 1.23 wt.% in dentin, 0.72 wt.% in bone) [10]. It is also required for osteoporosis prevention; it stimulates the growth and development of bones, and it influences vitamin D secretion [11]. Interest in the strontium substitution of calcium phosphates increased after strontium ranelate was found to enhance osteoblast differentiation, stimulate bone formation, and slow down the resorption activity and differentiation of osteoclasts, thus reducing the bone degradation rate [12,13,14]. 

Studies have shown that manganese plays an important role in maintaining bone structure and regulating bone metabolism [15,16,17]. Mn^2+^ ions increase the ligand-binding affinity of integrins, a large family of receptors which mediate cellular interactions with the extracellular matrix (ECM) and activate cell adhesion [18]. The in vitro and in vivo findings of Li et al. [19] revealed that Mn^2+^ ions released from Mn-containing bioceramics “inhibit the formation and function of osteoclasts, promote the differentiation of osteoblasts, and accelerate bone regeneration under osteoporotic conditions in vivo”. Many studies have focused on individual or dual-ion substitutions into the HAp crystal structures. However, the synthesis and characterization of multi-substituted hydroxyapatite have received less attention in the literature.

The major drawback of HAp scaffolds is their poor mechanical properties, especially their brittleness and low fracture toughness. Therefore, they cannot be used in conditions involving loads. To overcome these disadvantages, HAp has been combined with polymers [20] such as polycaprolactone (PCL), a biodegradable polymer widely used for the production of BTE scaffolds [21,22].

A very interesting strategy used to prepare biomimetic BTE scaffolds involves the use of marine organisms. The hard tissues of most marine organisms are made of calcium carbonate in the form of calcite or aragonite, which can be transformed into calcium phosphate ceramics [23]. For years, our research group has been systematically investigating the potential of cuttlebones for the preparation of highly porous HAp-based BTE scaffolds. Cuttlebone is an inexpensive, readily available, natural material that has a very high porosity (80~90%) and an interconnected pore structure with pore sizes suitable for cell attachment [24]. Additionally, the use of a biowaste material and its transformation into a value-added product, such as a BTE scaffold, is an eco-friendly and sustainable approach [25].

Our biomimetic strategy involves the hydrothermal transformation of cuttlefish bone (aragonite) into porous HAp ceramics, as well as the introduction of small amounts of trace ions, which are important for bone formation, into the hydroxyapatite structure.

In our previous studies, individual (Mg^2+^ and Sr^2+^) and dual-ion substitutions (Mg^2+^ and Sr^2+^) into the HAp crystal structure have been studied [26,27]. Mechanical (compression) tests were performed on uncoated and PCL-coated Mg and Mg-Sr substituted calcium-phosphate structures. The PCL coatings improved the Young’s modulus of all the investigated scaffolds, which increased more than five times compared to the uncoated scaffolds. The compressive strengths of the uncoated scaffolds ranged between 0.20 and 0.35 MPa, while the coated scaffolds showed values higher than 1.1 MPa. In vitro cell culture studies showed that scaffolds are nontoxic and provide an adequate 3D support for cell attachment and proliferation [26].

In the present work, we prepared scaffolds using various levels of manganese substitution, as well as a multi-substituted scaffold resulting from the simultaneous addition of two (Mg^2+^ and Sr^2+^) or three ions (Mn^2+^, Mg^2+^, and Sr^2+^) using the hydrothermal method. According to our knowledge, the hydrothermal preparation of Mn^2+^, Mg^2+^, and Sr^2+^ multi-substituted CaP scaffolds has not been performed thus far. The effects of ion substitutions on the microstructure, composition, and scaffolds’ surface morphology were analyzed. The multi-substituted CaP scaffold was coated with polycaprolactone using a simple vacuum impregnation technique. The human mesenchymal stem cells’ response upon culture on the 3D scaffold was characterized by histology, immunohistochemistry, and reverse transcription quantitative real-time polymerase chain reaction (RT-qPCR) analysis.

## 2. Experimental

### 2.1. Materials

Cuttlefish bones from the Adriatic Sea were used as the starting material for the hydrothermal synthesis of hydroxyapatite. An exterior dorsal shield was mechanically removed from the cuttlefish bones. The lamellae matrix of the bones was cut into pieces (≈2 cm^3^) and treated with an aqueous solution of sodium hypochlorite (NaClO, 13% active chlorine, Gram-mol, Zagreb, Croatia) at room temperature for 48 h to remove the organic component, and repeatedly washed with boiling demineralized water.

To synthesize non-substituted and substituted hydroxyapatites (the former being used as a reference sample) with compositions corresponding to the formula Ca_10-x_M_x_(PO_4_)_6_(OH)_2_, M = Mn, Mg, Sr), a procedure elaborated previously was followed [26,27]. In short, the required amounts of biogenic CaCO_3_ (in the form of cca. 2 cm^3^ pieces of cuttlefish bone), an aqueous solution of ammonium dihydrogenphosphate, as well as precursors of Mn^2+^, Mg^2+^, and Sr^2+^ ions were placed in a stainless steel container and kept in an oven for 48 h at 200 °C. Mn^2+^, Mg^2+^, and Sr^2+^ ions were added in the form of manganese(II) chloride tetrahydrate (MnCl_2_×4H_2_O, 99%, Acros Organics BV, Geel, Belgium), magnesium perchlorate (Mg(ClO_4_)_2_, 99%, Sigma Aldrich, Steinheim, Germany), and strontium nitrate (Sr(NO_3_)_2_, 99%, ACS reagent, Acros Organics, Geel, Belgium), respectively.

Manganese-substituted systems with a Mn/(Ca + Mn) molar ratio of 0.01, 0.025, 0.05 were prepared and designated as 1-Mn-HAp, 2.5-Mn-HAp, and 5-Mn-HAp, respectively. Furthermore, a Mg^2+^- and Sr^2+^- co-substituted system, corresponding to the nominal composition Ca_9_._8_ Mg_0_._1_Sr_0_._1_(PO_4_)_6_(OH)_2_, and a Mn^2+^-, Mg^2+^-, Sr^2+^-substituted system, corresponding to the composition Ca_9_._7_ Mn_0_._1_ Mg_0_._1_Sr_0_._1_(PO_4_)_6_(OH)_2_ were prepared as well, and designated as 1-Mg-1-Sr-HAp and 1-Mn-1-Mg-1-Sr-HAp, respectively. The non-substituted scaffold was designated as HAp.

### 2.2. Characterization Techniques

An XRD analysis was performed on powder samples using a Shimadzu XRD 6000 (Shimadzu Corporation, Tokyo, Japan) diffractometer with Cu Kα radiation, with a set addition of silicon standard, as described previously [26]. The scanning was performed in the 2*θ* range between 5° and 70°, with the step size set to 0.02°. The whole powder pattern decomposition refinements were carried out to determine the lattice parameters, the average crystallite sizes, and the constituting phase weight percentages using TOPAS software (version 5.0, Bruker, Karlsruhe, Germany) [28]. Structures of calcium phosphates reported in [29,30] were used as starting models for the refinements.

FTIR analyses were conducted using a Bruker Vertex 70 spectrometer (Bruker Optik GmbH, Ettlingen, Germany) equipped with a diamond crystal attenuated total reflectance (ATR) unit. The spectra were collected in the wave number range of 400 to 4000 cm^−1^, with the spectral resolution set to 4 cm^−1^ for an average of 32 scans.

A scanning electron microscope (SEM Tescan Vega III Easyprobe, Tescan Orsay Holding, Brno, Czech Republic) was used to analyze the scaffolds’ surface morphology after gold and palladium sputtering.

### 2.3. Preparation of Composite Scaffolds

Polycaprolactone (PCL, Mn = 45,000, Sigma-Aldrich, Steinheim, Germany) was dissolved in chloroform (p.a., Kemika, Zagreb, Croatia). Scaffold specimens 1-Mn-1-Mg-1-Sr-HAp were degassed in a vacuum chamber (CitoVac, Struers, Cleveland, OH, USA) and then soaked in the PCL solution (10% *w*/*v*). The detailed experimental procedure is described in [26].

### 2.4. Stem Cell Studies

hMSCs cells were kindly provided by Prof. Inga Urlić, University of Zagreb Faculty of Science, Zagreb, Croatia. Bone marrow was collected during a surgery at the University Hospital of Traumatology in Zagreb, Croatia, with the informed consent of the patient. All procedures were approved by the Ethics Committee. The primary isolation of hMSCs from the bone marrow aspirates and the cell culture of hMSCs on prepared scaffolds under static conditions were performed as described in detail earlier [31,32]. hMSCs cells were seeded (5 × 10^5^ cells/200 μL of proliferation medium per well) onto sterilized PCL-coated 1-Mn-1-Mg-1-Sr-HAp scaffolds and incubated at 37°C and 5% CO_2_ for 24 h. The seeded scaffolds were cultured for three weeks in the osteogenic medium “containing Minimum Essential Medium-Alpha Eagle (*α*-MEM, Lonza, Basel, Switzerland), 10% FBS, 1% penicillin/streptomycin, 50 μg/mL ascorbic acid (Sigma-Aldrich, Steinheim, Germany), 4 mmol/L *β*-glycerophosphate (Sigma-Aldrich, Steinheim, Germany) and 1 × 10^7^ mol/L dexamethasone (Sigma-Aldrich, Steinheim, Germany)” [26]. The medium was changed every 2 days for three weeks. The scaffolds were processed for hematoxylin and eosin staining, as described in [26]. “The scaffolds with cells were embedded in paraffin, Biowax blue (BioGnost, Zagreb, Croatia) and cut into 5 μm thick sections using a rotary microtome (Esselite Leitz, Stuttgart, Germany). After deparaffinization, the slides were stained with hematoxylin and eosin for 2 min. Slides were mounted with coverslips and observed by light microscopy (Olympus BX51, Shinjuku, Tokyo, Japan)”.

The immunohistochemical detection of collagen type I was carried out as described in our previous works [26,27]. In short, after 21 days of culture, the scaffolds were removed from the osteogenic medium, fixed in 4% paraformaldehyde, transferred into a PBS buffer solution with 30% sucrose for 48 h, embedded in a compound for cryosectioning (Tissue-Tek O.C.T. compound, Sakura, CA, USA), and frozen at −80 °C. The blocks were cut into 10 μm thick sections using a cryostat microtome (Leica, Wetzlar, Germany). The sections were incubated with primary antibody (anti-collagen I, Abcam, Cambridge, UK). Immunohistochemical staining was performed using the EnVision ™ Detection System Peroxidase/DAB + , Rabbit/Mouse (Dako, Glostrup, Denmark), according to the manufacturer’s instructions. Scaffolds without cells but in the same culture condition, were used as blanks. Negative controls were processed in the same way, albeit with the primary antibody omitted. Human bone was used as the positive control. All slides were visualized using an Olympus BX51 microscope, and images were captured using a digital camera.

The expression of levels of osteoblast genes, alkaline phosphatase (ALP), and dentin matrix protein 1 (DMP1) was quantitatively determined at day 21 of culture by real-time quantitative polymerase chain reaction (RT-qPCR), as described in our previous work [26]. In short, the total RNA was extracted using the TRIzol reagent (Invitrogen Life Technologies, Sigma-Aldrich, Steinheim, Germany) according to the manufacturer’s instructions. The expression of the target genes was quantitatively determined using the 7500 Fast Real-Time PCR System (Applied Biosystems, Thermo Fisher Scientific, Waltham, MA, USA) using a Power SYBR green PCR master mix (Applied Biosystems, Thermo Fisher Scientific, Waltham, MA, USA). All genes were normalized to *β*-actin, and relative expression levels were calculated using the ∆∆Ct method.

**Scheme 1:**
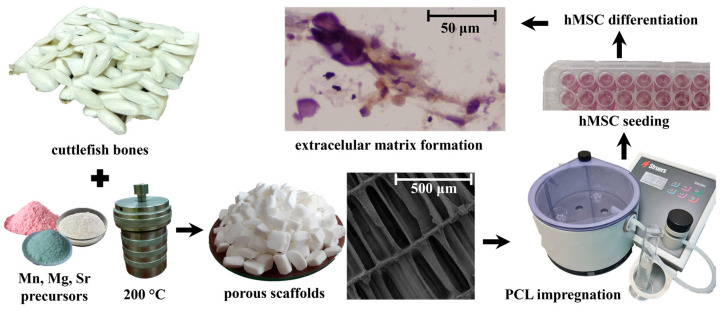
An illustration of experimental procedure.

The experimental procedure is illustrated in Scheme 1.

### 2.5. Statistical Analysis

The statistical significance between different groups was examined by applying a one-way ANOVA test, with *p* < 0.05 and *p* < 0.01 being considered statistically significant.

## 3. Results

### 3.1. XRD Analysis

**Figure 1:**
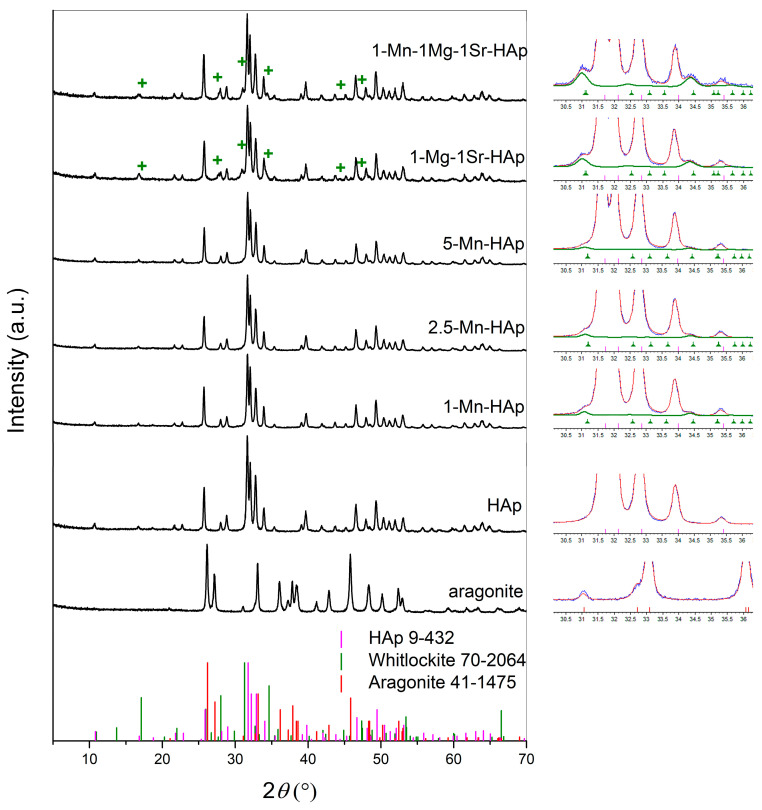
Left column: X-ray diffraction patterns of synthesized materials and the standard XRD lines of hydroxyapatite (ICDD 09-432), whitlockite (ICDD 70-2064), and aragonite (ICDD 41-1475). Right column: the deconvolution of the XRD pattern by the Rietveld method. The blue line is the recorded experimental XRD pattern, the red line is the calculated XRD pattern of the entire sample, and the green line corresponds to the contribution of whitlockite.

The powder X-ray diffraction patterns of synthesized materials are shown in Figure 1, including the XRD spectrum of aragonite cuttlefish bone. The non-substituted material (HAp) shows well-defined reflections, which is in accordance with the standard XRD lines of hydroxyapatite (ICDD 09-432). In ion-substituted samples, in addition to hydroxyapatite, whitlockite was also detected (based on ICDD 70-2064). As seen, there is no significant difference between the XRD patterns of the substituted and non-substituted scaffolds, suggesting that the lattice structure of HAp is not markedly affected by ion substitutions. The whitlockite peaks in the XRD patterns of 1-Mg-1-Sr-HAp and 1-Mn-1-Mg-1-Sr- are designated by + (Figure 1, left column). The whitlockite peaks are hardly visible in the XRD patterns of Mn-HAps due to its very low content. The deconvolution of the XRD pattern by the Rietveld method, showing the contribution of whitlockite (green line), are illustrated in the right column of Figure 1. The blue line is the recorded experimental XRD pattern, while the red line is the calculated XRD pattern of the entire sample.

The whole powder pattern decomposition refinement of the XRD data was applied for the quantitative phase analysis, the evaluation of lattice parameters, and the crystallite size of hydroxyapatite, as shown in Table 1. The refinements’ quality was checked from the resultant weighted profile factors (Rwp). The values of Rwp were between 6.2 and 7.3 (Table 1), indicating a good accuracy of the refinements. The results of quantitative analysis did not indicate the presence of non-transformed aragonite in any of the scaffolds.

As seen in Table 1, the addition of 1 to 5 mol % of Mn^2+^ did not significantly influence the phase composition of the prepared scaffolds, although it seems that an increased Mn^2+^ content resulted in a slightly higher content of the hydroxyapatite phase at the expense of the amount of the amorphous phase. A low amount (cca. 2%) of the whitlockite (WH) phase, typical for Mg-substituted calcium phosphates, was also detected in the Mn-substituted systems.

The scaffold 1-Mn-1-Mg-1-Sr-HAp, as a result of the simultaneous addition of the equimolar amount (1 mol%) of three ions (Mn^2+^, Mg^2+^, and Sr^2+^), showed the lowest content of the hydroxyapatite phase (67%) and the highest content of the amorphous (23%) and the whitlockite phase (10%) among the investigated systems.

**Figure 2:**
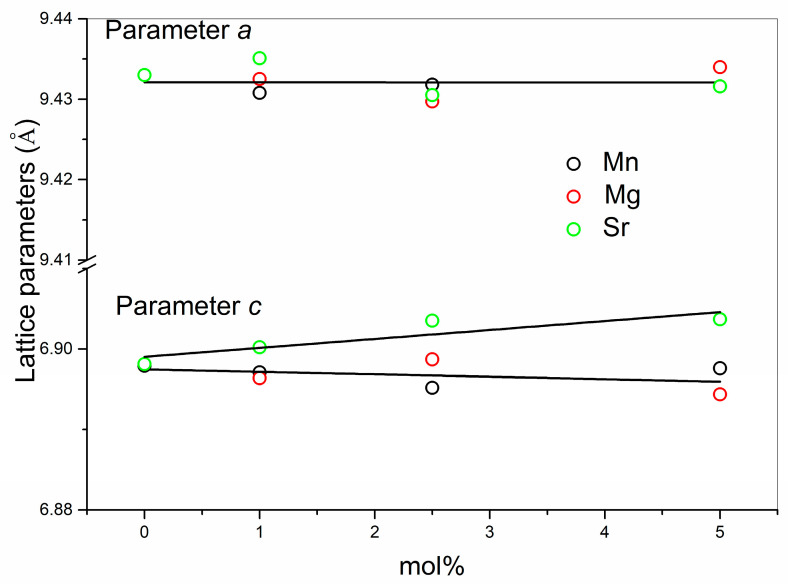
Lattice parameters *a* and *c* of the hydroxyapatite phase as a function of the mol% of the substituents.

As seen in Table 1, values of the hydroxyapatite lattice parameters (*a* and *c*) in the prepared Mn-substituted systems are smaller than those of the non-substituted system, which is expected considering the smaller radius of Mn^2+^ compared to Ca^2+^. The HAp parameters of the Mn-substituted systems were compared to those of the Mg-substituted and the Sr-substituted systems. Figure 2 shows lattice parameters *a* and *c* of the hydroxyapatite phase as a function of the mol % of the substituents obtained in this (Mn) as well as our previous studies (Mg and Sr) [26,27].

For the Mg- and Sr- substituted systems, no significant change in the lattice parameter *a* of the hydroxyapatite phase was observed, while parameter *c* shows a slight linear decrease with increased levels of Mg^2+^ and a linear increase with increased levels of Sr^2+^, which coincides with the smaller radius of Mg^2+^ and the larger radius of Sr^2+^ with respect to Ca^2+^. For the lowest 1 mol % substitutions, very similar values of the parameters were observed for both Mg^2+^- and Mn^2+^-substituted HAps.

### 3.2. FTIR Analysis

**Figure 3:**
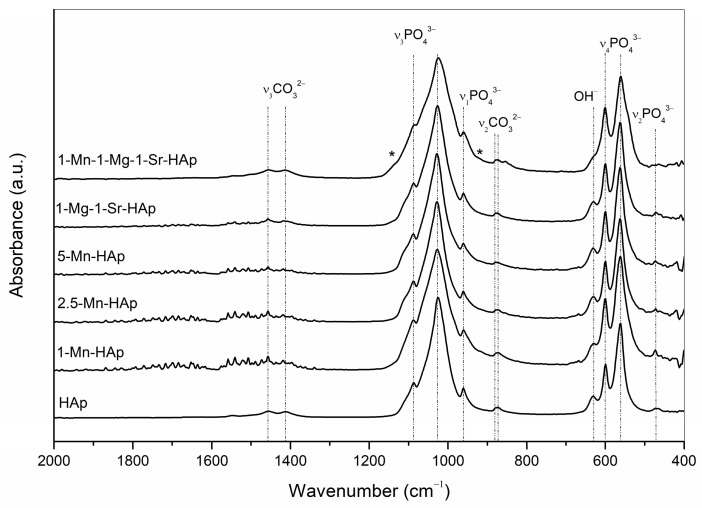
FTIR spectra of synthesized materials. The appearance of low-intensity vibrational bands characteristic for whitlockite are marked with *.

The normalized FTIR spectra of the synthesized materials are shown in Figure 3. No significant differences in the characteristic absorption frequencies were seen among the samples, although the spectra of substituted scaffolds show a slight broadening compared to the non-substituted scaffold. For easier comparison, the observed FTIR band positions and their assignments are summarized in Table 2.

The most intensive bands at 1085–1088 cm^−1^ and 1024–1028 cm^−1^ can be assigned to antisymmetric vibrations (*ν*_3_) of P-O bonds. The bands at 600–601 cm^−1^ and 561–563 cm^−1^ are due to bending vibrations (*ν*_4_) of O-P-O groups. The band at 960 cm^−1^ is assigned to symmetric vibrations (*ν*_1_) of P-O bonds. The band located at 472–473 cm^−1^ is assigned to the *ν*_2_ bending vibration of PO_4_^3−^ units. The band in the region 629–632 cm^−1^ is derived from the OH^−^ vibrations. The bands located at 872–873 cm^−1^ and 876–879 cm^−1^ were detected in all spectra and are assigned to *ν*_2_ vibration modes of the CO_3_^2−^ group. For non-substituted and co-substituted systems, the carbonate *ν*_3_ bands at 1413, 1456, and 1546–1549 cm^−1^ were detected. For Mn-substituted samples, the carbonate *ν*_3_ bands were not possible to determine due to pronounced noise. It is generally accepted that bands at 1546, 1456, and 880 cm^−1^ are the characteristic bands of CO_3_^2−^ substitution for OH^−^ in the apatite lattice (type A substitution), while the 1465, 1413, and 873 cm^−1^ bands indicate CO_3_^2−^ substitution for PO4^3−^ (type B substitution). The band at 1465 cm^−1^ which is characteristic of a type B substitution is not seen, and it is eventually masked in the shoulder of the band at 1456 cm^−1^. The weak bands (peaks/shoulders) at 1134 and 922 cm^−1^ (marked with * in Figure 3) indicate whitlockite formation [33], consistent with the XRD results.

### 3.3. SEM Analysis

**Figure 4:**
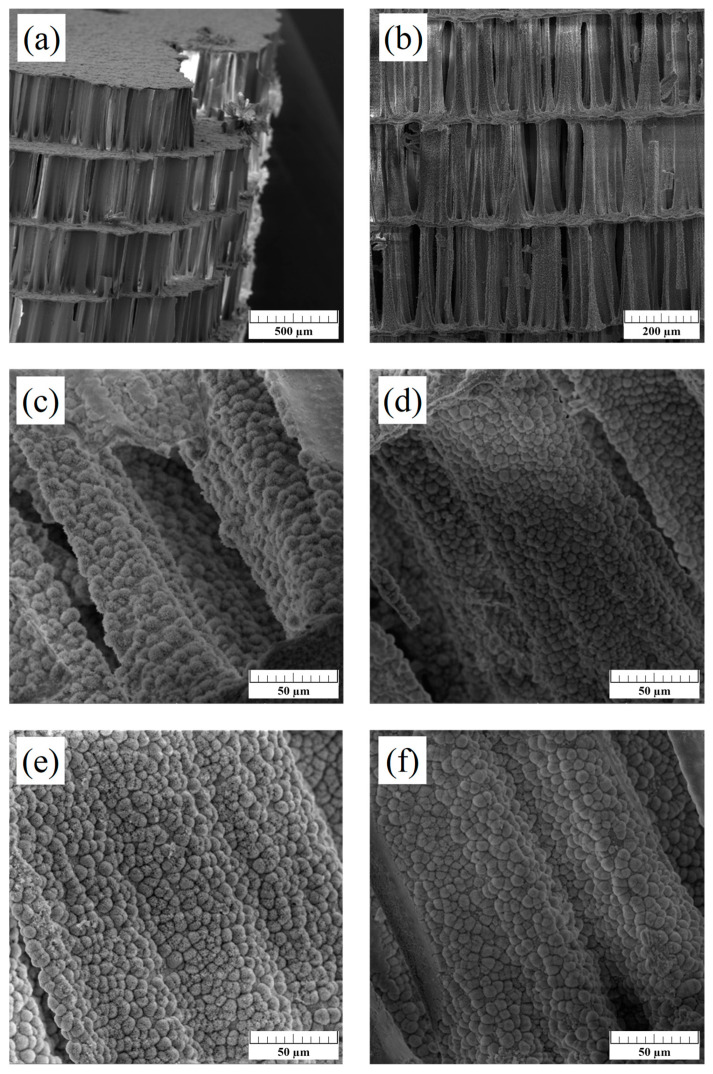
SEM micrographs displaying interconnected structure (**a**,**b**) and surface morphology of 1-Mn-HAp (**c**), 2.5- Mn-HAp (**d**), 5- Mn-HAp (**e**), and 1-Mn-1Mg-1-Sr-HAp (**f**) scaffolds. Scale bar: 500 μm (**a**), 200 μm (**b**) 50 μm (**c**–**f**).

As seen from the SEM micrographs (Figure 4), the initial porous cuttlebone structure was preserved after hydrothermal synthesis, which is crucial for bone tissue applications, allowing for the migration of cells into the scaffold and the diffusion of nutrients to cells. The SEM analysis has shown that all prepared scaffolds have the same surface morphology despite differences in ion concentration or simultaneous substitution of different ions (Figure 4c–f). The size of hydroxyapatite spheres, forming a “cauliflower-like morphology”, is similar to those reported in a previous study [34].

**Figure 5:**
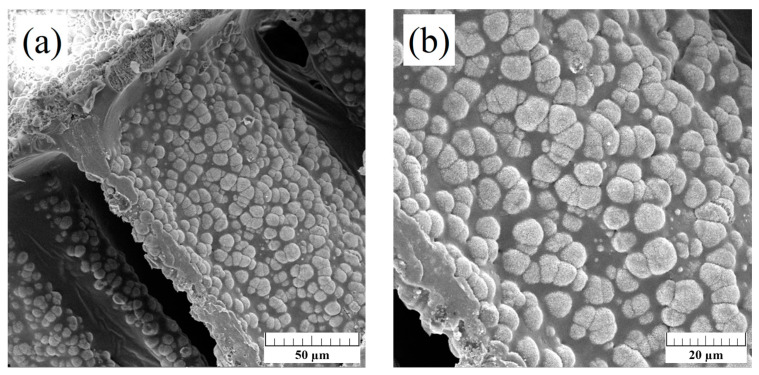
SEM micrographs of PCL-coated 1-Mn-1-Mg-1-Sr-HAp scaffold, at two different magnifications. Scale bar: 50 μm (**a**); 20 μm (**b**).

SEM images of PCL-coated 1-Mn-1-Mg-1-Sr-HAp scaffold, at two different magnifications, are given in Figure 5, showing that polymer coating did not compromise the interconnections of the pores. The surface roughness due to hydroxyapatite microspheres is still present, which is important for cell adhesion and proliferation [35].

### 3.4. Stem Cell Studies

**Figure 6:**
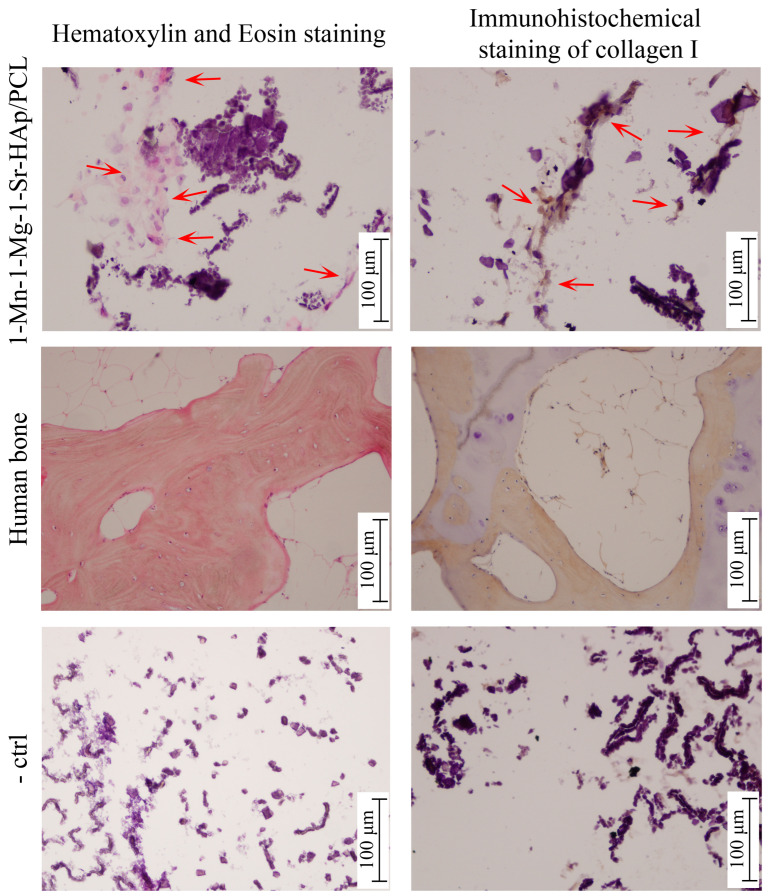
Hematoxylin/eosin and immunohistochemical staining of hMSCs cultured for three weeks on the PCL-coated 1-Mn-1Mg-1-Sr-HAp scaffold in an osteogenic medium. Red arrows point to stained regions where a bone-like matrix is forming. Human bone served as positive control, and the scaffold without cultured cells served as negative control (-ctrl).

Results of hematoxylin and eosin (H&E) staining and immunohistochemical staining of collagen type I are shown in Figure 6. The negative control sections of each sample (-crtl, Figure 6) represent staining interference originating from scaffold material and should be excluded during the comparison. Dark purple areas in Figure 6 originate from the HAp-based scaffold. The optical image of the cross-section of the 1-Mn-1-Mg-1-Sr-HAp/PCL scaffold stained with H&E shows pink-colored areas (marked by arrows), indicating the formation of a bone-like matrix, which is consistent with the immunohistochemical staining results. Brown staining (Figure 6, right column, marked by arrows) indicates collagen I expression, forming the extracellular matrix (ECM) of the interconnected network structure.

**Figure 7:**
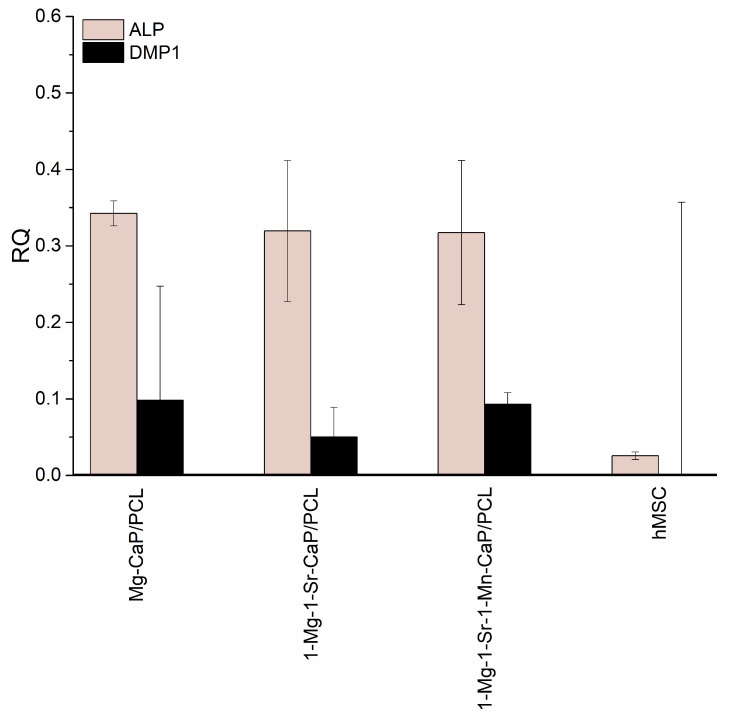
Relative gene expression of hMSCs cultured on different scaffolds for three weeks in osteogenic medium, quantified by RT-PCR, normalized to β-actin.

A quantitative RT-PCR analysis showed that the expressions of alkaline phosphatase (ALP) and dentin matrix protein 1 (DMP1), after three weeks of hMSCs culture on the PCL-coated 1-Mn-1Mg-1-Sr-HAp scaffold in an osteogenic medium, are comparable to the levels detected previously [26] in hMSCs cultured on PCL- coated scaffolds 1-Mg-CaP/PCL and 1-Mg-1-Sr-CaP (Figure 7).

## 4. Discussion

In this study, Mn-substituted scaffolds, with various levels of manganese substitution for Ca into hydroxyapatite (1, 2.5, and 5 mol%), a Mg- and a Sr-co-substituted scaffold corresponding to the nominal composition Ca_9_._8_ Mg_0_._1_Sr_0_._1_(PO_4_)_6_(OH)_2_ (1-Mg-1-Sr-HAp), and a multi-substituted scaffold (1-Mn-1-Mg-1-Sr-HAp), as a result of the simultaneous addition of an equimolar amount (1 mol%) of three ions (Mn^2+^, Mg^2+^, and Sr^2+^), were prepared. Non-substituted scaffolds were prepared as a reference.

The analysis of the XRD data indicates that the non-substituted system is a biphasic mixture composed of hydroxyapatite (HAp) and the amorphous phase, while all ion-substituted samples were identified as a triphasic mixture, composed of hydroxyapatite, whitlockite (WH), and the amorphous phase. The results of a quantitative analysis of samples obtained through the whole powder pattern decomposition of XRD data do not indicate the presence of non-transformed aragonite in any scaffold. The presence of WH and amorphous calcium phosphate (ACP), which are more soluble than HAp in physiological conditions, can enhance bone repair [27,36]. As reported previously [37], the presence of whitlockite in HAp/WH scaffolds significantly promoted the osteogenic activity of hMSCs. The amount of HAp in the Mn-substituted scaffolds (between 82 and 86 wt.%) and in the 1Mg-1Sr-HAp scaffold (84 wt.%) is comparable to HAp wt.% in the non-substituted scaffold (83 wt.%). Increased Mn content resulted in a slight decrease in the relative amount of the amorphous phase (from cca. 16 wt.% in 1Mn-HAp to 13wt.% in 5-Mn-HAp), which is opposite to the results of Bracci et al. [38], who found that the relative amount of the amorphous phase increased as a function of Mn^2+^ concentration. Bracci et al. prepared apatite powders using the precipitation method at 37 °C and concluded that Mn^2+^ ions hinders the synthesis of HAp and promotes the precipitation of an amorphous Ca–Mn phosphate. The different reaction conditions might be the reason for the opposite results. A low amount (cca. 2 wt.%) of the WH phase, typical for Mg-substituted calcium phosphates, was also observed in the Mn-substituted systems. Since the ionic radii of Mg^2+^ and Mn^2+^ are nearly identical, it can be assumed that the whitlockite structure can accommodate Mn^2+^ ions as well. In the 1-Mg-1-Sr-HAp scaffold, cca. 8 wt.% of the WH phase and cca. 9 wt.% of the amorphous phase were estimated, while the 1-Mn-1-Mg-1-Sr-HAp scaffold showed the lowest content of the hydroxyapatite phase (67 wt.%) and the highest content of the amorphous (wt. 23%) and the whitlockite phase (10 wt.%) among the investigated systems.

Lattice parameters *a* and *c* and the unit-cell volume of the hydroxyapatite phase (Table 1) in the prepared Mn-substituted systems are smaller than those of the non-substituted system, which is consistent with the smaller radius of Mn^2+^ compared to Ca^2+^, indicating the presence of Mn^2+^ in the HAp structure. However, the sample 5-Mn-HAp violates the linear decrease in the parameter *c* with increased level of Mn, suggesting that a very limited quantity of Mn^2+^ ions was incorporated into the HAp structure. The main portion of Mn^2+^ ions could be surface-bound or present in the amorphous phase, as reported by Bracci et al. [38]. Kurunakara et al. [39] also reported that Mn doping up to 10 mol% did not affect the HAp structure. For the lowest 1 mol% substitutions (1-Mn-HAp scaffold) the values of parameters *a* and *c* of the hydroxyapatite lattice are very similar to those estimated for the corresponding Mg-substituted HAp [27].

In our previous studies [26,27] we performed hydrothermal syntheses of non-substituted, Mg^2+^-substituted, Sr^2+^-substituted, and Mg^2+^- and Sr^2+^-co-substituted calcium phosphate-based scaffolds. Due to the natural variability in biological systems such as a cuttlefish bone (the source of calcium ions), in this study, we repeated the preparation of non-substituted (HAp) and Mg- and Sr-co-substituted (1-Mg-1-Sr-HAp) systems, and compared the microstructural results to those determined in our previous studies [26,27]. The lattice parameters determined for the non-substituted hydroxyapatite prepared in this study (HAp, Table 1) have identical or very similar values to those determined in our previous study [27] (where *a* = 9.4330 Å; *c* = 6.8981 Å; and cell volume = 531.57 Å^3^), suggesting no significant chemical variability in the biological source of Ca ions used (cuttlefish bones). Also, an excellent agreement between the HAp parameters of the Mg-Sr-co-substituted scaffold prepared in the present study (Table 1, 1-Mg-1-Sr-HAp) and those estimated in our previous study [26] (where *a* = 9.4317 Å; *c* = 6.9014 Å; cell volume = 531.674 Å^3^; and crystallite size = 62.1 nm) was observed. This is important to know when analyzing the influence of Mn ions on the microstructure of hydroxyapatite obtained in the presence of all three ions in the reaction mixture.

As seen in Table 1, values of lattice parameters of 1-Mn-1-Mg-1-Sr-HAp are higher compared to 1-Mg-1-Sr-HAp. Since only Sr^2 +^ ions tend to increase the lattice parameters, it seems that Sr^2 +^ ions can be more easily incorporated into the hydroxyapatite lattice if they are in the presence of Mn^2+^ ions. Our results are opposite to those reported by da Silva et al. [40], who found that “the capture of Sr^2+^ during the precipitation process was less favorable than the capture of Mg^2+^ and Mn^2+^ when they were present at the same concentration in the reaction medium”.

In the FTIR spectra of non-substituted and co-substituted systems, the carbonate *ν*_3_ bands at 1413, 1456, and 1546–1549 cm^−1^ were detected. It is generally accepted that bands at 1546, 1456, and 880 cm^−1^ are the characteristic bands of CO_3_^2−^ substitution for OH^−^ in the apatite lattice (type A substitution), while the 1465, 1413, and 873 cm^−1^ bands indicate CO_3_^2−^ substitution for PO4^3−^ (type B substitution). According to the results of Ren et al. [41], the bands at 880, 1413, and 1450 cm^−1^ should not be used as characteristic bands of carbonated apatites since they could result from carbonate adsorbed onto the apatite crystals’ surface. The combined experimental and computational study [41] revealed that the carbonate *v_3_* band at 1546 cm^−1^ is the characteristic band for type A carbonated apatites, and that the 1465 cm^−1^ band is characteristic for type B carbonated apatites. Our FTIR results indicate type A carbonate substitution. In terms of chemical composition, carbonated hydroxyapatite is more similar to the natural bone mineral than stoichiometric hydroxyapatite [42], showing an enhanced biocompatibility [43,44]. Therefore, produced scaffolds may have potential as bone substitutes.

The initial porous cuttlebone structure was preserved after hydrothermal transformation to calcium phosphate. In the hydrothermal synthesis performed in this study, an insoluble calcium-based precursor (CaCO_3_, aragonite) was used, which was immersed in an aqueous phosphate solution. In the aqueous solvent, which is above its normal boiling temperature and pressure, the calcium material undergoes a slow and gradual dissolution and recrystallization at the solution/calcium source interface through continuous nucleation-growth-ripening steps [45]. By the nucleation/precipitation of HAp crystals on the surface of dissolving CaCO_3_, the original material is replaced, and the initial porous structure of scaffold is preserved.

No significant influence of Mn^2+^ ion concentration or simultaneous substitution of different ions on the scaffolds surface morphology was observed by SEM (Figure 4). A “Cauliflower-like morphology”, characteristic for hydroxyapatite, was observed on the surface of “lamellae and pillars” of the scaffolds, similar to those reported in a previous study [34]. The calculated crystallite size ranges from 46 to 62 nm (Table 1) and is within the order of magnitude of nano-sized crystals in bones.

The differentiation of human mesenchymal stem cells (hMSCs), cultured on the PCL-coated 1-Mn-1-Sr-1-Mg-HAp scaffold in an osteogenic medium for three weeks, into osteoblasts was evaluated. One of the earliest stages in hMSC osteogenic differentiation is the production of type I collagen [46] that can stimulate osteogenic differentiation without additional osteoinductive factors [47]. Collagen I synthesis was confirmed by positive immunohistochemical staining (Figure 6). Hematoxylin/eosin staining also indicated the formation of a bone-like matrix. The quantitative RT-PCR analysis showed the expression of osteogenic genes, alkaline phosphatase (ALP), and dentin matrix protein 1 (DMP1) after three weeks of hMSC s culture. ALP is one of the earliest markers of osteoblastic cell differentiation [48], while DMP1 plays an essential role in bone mineralization [49]. The levels of ALP and DMP1 expression in hMSCs cultured on PCL- coated 1-Mn-1-Mg-1-Sr-HAp scaffold are comparable to the levels detected previously [26] in hMSCs cultured on PCL-coated 1-Mg-CaP/PCL and 1-Mg-1-Sr-CaP scaffolds (Figure 7), where the higher expression of ALP compared to DMP1 expression indicated the retention of hMSCs at an early stage of osteogenic differentiation. It is worth noting that the RT-qPCR analysis of uncoated CaP and Mg-CaP scaffolds [27] showed a higher expression of DMP1, compared to the expression of ALP, which is indicative of the later stages of osteoinduction. The overall results indicate that the PCL coating might have a dominant influence on cell behavior, at least in the investigated period of cell culture time. It can be assumed that PCL coating hinders or even impedes ion release from substituted calcium phosphate structures and thus reduces their positive effect on the osteogenic differentiation of hMSCs. This assumption should be addressed in future studies. Further in vitro studies on ion release are needed to investigate the long-term behavior of prepared composite scaffolds. To confirm its suitability for bone tissue engineering applications, in vivo studies on animal models need to be performed as well.

## 5. Conclusions

A range of porous calcium phosphate (CaP) scaffolds substituted with one (Mn^2+^), two (Mg^2+^ and Sr^2+^-), and three (Mn^2+^, Mg^2+^, and Sr^2+^-) ions were prepared by the hydrothermal conversion of cuttlefish bone. The simultaneous addition of an equimolar amount (1 mol%) of the three ions resulted in a lower content of the hydroxyapatite phase (67%) and a higher content of the amorphous (23%) and the whitlockite phase (10%), when compared to unsubstituted, Mn-substituted, and Mg- and Sr-co-substituted CaP systems. The presence of a more soluble whitlockite and amorphous phase could be beneficial for bone repair. The human mesenchymal stem cells’ (hMSCs) response upon culture on the PCL coated Mn^2+^-, Mg^2+^-, Sr^2+^-substituted scaffold was followed by histology, immunohistochemistry, and RT-qPCR analysis. The level of the alkaline phosphatase (ALP) and the dentin matrix protein 1 (DMP1) expression of the 1-Mn-1-Mg-1-Sr-HAp/PCL scaffold is comparable to the expression from hMSCs cultured previously on the 1-Mg-CaP/PCL and 1-Mg-1-Sr-CaP/PCL scaffolds, indicating that the PCL coating might have a dominant influence on cell behavior, at least in the investigated period of cell culture time. Further in vitro studies of ion release are needed to investigate the long-term behavior of the prepared composite scaffold. To confirm its suitability for bone tissue engineering applications, in vivo studies on animal models need to be performed as well.

## Figures and Tables

**Table 1:** Results of phase quantification and the crystallographic parameters of hydroxyapatite phase.

	HAp	1-Mn- HAp	2.5-Mn- HAp	5-Mn- HAp	1-Mg-1-Sr- HAp	1-Mn-1-Mg-1-Sr-HAp
Hydroxyapatite, wt.%	82.55	82.05	84.92	85.77	83.54	67.33
Whitlockite, wt.%	0	2.00	1.92	1.62	7.65	9.88
Amorphous phase, wt.%	17.45	15.95	13.16	12.61	8.81	22.79
R_wp_	6.222	6.222	6.953	6.937	6.788	7.325
HAp parameters						
*a*, Å	9.4330	9.4308	9.4318	9.4316	9.4316	9.4333
*c*, Å	6.8979	6.8971	6.8952	6.8976	6.9014	6.9042
Cell volume (Å^3^)	531.55	531.24	531.21	531.37	531.67	532.07
Crystallite size, nm	47.4	48.4	58.7	61.7	61.4	46.5

**Table 2:** Observed FTIR band positions and their assignments.

	PO_4_^3−^				CO_3_^2−^		OH^−^
Code	ν_2_	ν_4_	ν_1_	ν_3_	ν_2_	ν_3_	
HAp	472	561 600	960	1024 1088	873 877	1413 1456 1549	632
1-Mn-HAp	473	563 600	960	1028 1088	872 876		629
2.5-Mn-HAp	473	563 600	960	1028 1088	872 877		631
5-Mn-HAp	473	563 600	960	1028 1088	873 879		631
1-Mg-1-Sr-HAp	472	563 600	960	1026 1087	873 878		631
1-Mn-1-Mg-1-Sr-HAp	473	561 601	960	1024 1085	873 877	1413 1456 1546	

## Data Availability

Data are contained within the article.
